# Fusarioid keratitis and other superficial infections: A 10-years prospective study from Northeastern Brazil

**DOI:** 10.1371/journal.pntd.0012247

**Published:** 2024-06-17

**Authors:** Jose Ferreira da Cunha Neto, Walicyranison Plinio da Silva Rocha, Georgios Makris, Marcelo Sandoval-Denis, Ferry Hagen, Pedro Willem Crous, Guilherme Maranhão Chaves

**Affiliations:** 1 Laboratory of Medical and Molecular Mycology, Department of Clinical and Toxicological Analyses, Federal University of Rio Grande do Norte, Natal, Rio Grande do Norte, Brazil; 2 Department of Pharmaceutical Sciences, Federal University of Paraiba, João Pessoa, Paraíba, Brazil; 3 Westerdijk Fungal Biodiversity Institute, Utrecht, the Netherlands; Albert Einstein College of Medicine, UNITED STATES

## Abstract

**Background:**

*Fusarium* and allied genera (fusarioid) species are common colonizers of roots and aerial plant parts, or act as phytopathogens in forestry and horticultural or grain crops. However, they can also cause a wide range of infections in humans, including onychomycosis, cutaneous and invasive infections. Fusarioid keratitis is characterized by an infection of the cornea with a suppurative and ulcerative appearance, which may cause damage to vision and permanent blindness. The aim of the present study was to investigate the prevalence of fusarioid species, biofilm formation and antifungal susceptibility profiling of clinical isolates recovered from patients with keratitis and dermatomycoses.

**Methodology/Principal findings:**

The study was performed between March, 2012-December, 2022. Demographic, clinical and epidemiological data of patients were also collected. In the present study, most of the patients with keratitis were male (74%), had a median age of 42 years old, worked with plant material or debris and 26% of them reported eye trauma. Regarding dermatomycosis, most of patients were female and exhibited toenail lesions. Forty-seven isolates belonged to the genus *Neocosmospora* (78.33%), nine to the *Fusarium fujikuroi* (15%) and four to the *Fusarium oxysporum* (6.66%) species complexes. Several strains were moderate biofilm producers, specifically among *Fusarium annulatum*. Most strains showed increased MICs to amphotericin B and ketoconazole and low MICs to itraconazole. MICs ranged from 0.25 to 16 μg/mL for amphotericin B, 0.0625 to >16 μg/mL for ketoconazole and 0.125 to 8 for itraconazole.

**Conclusions/Significance:**

It is possible to conclude that fusarioid keratitis in Northeastern Brazil is an important and neglected disease, given the high number of cases, increased need for keratoplasty and poor outcome of the disease.

## Author summary

Fusariosis is an infectious disease caused by fungi belonging to fusarioid species. They are called “fusarioid” because there are other genus genetically related to *Fusarium*, which may cause the same disease. These microorganisms can be found in nature, specifically in different hosts, including agri-food and ornamental plants, forest trees, the soil and decomposing wood. Nevertheless, *Fusarium* spp. can cause diseases in vegetables, being considered phytopathogens. They can also cause infections in domestic animals such as dogs and horses as well as in seahorses, turtles, dolphins and pinnipeds. In humans, and in severely immunocompromised patients, fusariosis is the second most common systemic fungal infection, after invasive pulmonary aspergillosis. In addition to systemic infections, these fungi can cause superficial infections, such as dermatomycosis (mainly onychomycosis) and keratitis. Keratitis is an infection of the eye that may lead to blindness. In developed countries, it is mostly related to poor contact lens cleaning. However, in developing and tropical countries like Brazil, the most common source of contamination is through trauma of the eye, especially by plant material containing fungal spores. Therefore, this is a disease essentially related to poor agriculture workers. Therefore, we decided to have a look how keratitis and other fusarioid infections affect this specific population in the lowest income region of my country. We also investigated biofilm (organized fungal communities important for pathogenicity) formation in the strains recovered from these patients and their genetic relatedness. Another question that we wanted to answer was which antifungal drugs could be used to treat them.

## Introduction

Fusariosis is an infectious disease of worldwide distribution, caused by *Fusarium* spp. and related genera (fusarioid fungi) [1]. The term “fusarioid” fungi refers to fungi not belonging to the *Fusarium* genera, but possess fusarium-like macroconidia [2]. In fact, *Fusarium* is able to cause diseases in different living beings, including humans, other animals and plants. Therefore, these fungi have been recognized as “trans-kingdom” pathogens [3,4]. In humans, fusariosis may range from superficial (including keratitis, nail and skin infections) to life-threatening disseminated diseases, specifically among immunocompromised patients (neutropenic, leukemic patients, those under intense chemo or corticosteroid therapy, and transplant recipients) [5–8].

Keratitis is one of the most frequent diseases caused by fusarioid fungi and it is mostly related to trauma with plant debris or other organic matters, contact lens wear, ocular diseases and surgeries, mainly associated with the use of steroids [9,10]. Clinical signs of fusarioid keratitis may involve a grayish-white ulcer accompanied by peripheral feathery corneal infiltrate, endothelial plaque formation and hypopyon [11]. Fusarioid keratitis may progress to endophthalmitis or even cause corneal perforation and descemetocele, and in several occasions corneal transplantations and other surgeries are required [11–14].

Onychomycosis and other dermatomycosis represent important clinical entities caused by fusarioid spp. Although not considered life-threatening diseases, they may cause embarrassment and working-related challenges [15,16]. Onychomycoses due to fusarioid spp. almost always occur on toenails, especially after traumatic and dystrophic abnormalities or even subsequent to dermatophyte onychomycosis [17]. Risk factors for fusarioid onychomycosis may involve traumatic injury, diabetes mellitus, arterial hypertension, HIV and autoimmune illness [18]. It is worth mentioning that in case of immunosuppression, nail and other skin infections caused by fusarioid spp. may become disseminated [19].

It has been previously reported that fusarioid spp. may produce biofilms, and this structure has been associated with contact lenses keratitis outbreaks, most often related to the contamination of lens solution [20–22]. Biofilm formation in *Neocosmospora* (formerly the *Fusarium solani* species complex-FSSC) may vary among different strains, but usually consists of conidia, and a highly coordinated network of hyphal structures and an extracellular matrix (ECM) [21–23]. It is important to emphasize that sessile cells of biofilm-forming *Fusarium* strains exhibit elevated minimum inhibitory concentrations (MICs) for amphotericin B (AMB), voriconazole (VRC), and posaconazole, when compared to their planktonic counterparts [24].

Fusarioid spp. may be intrinsically resistant to a few antifungal drugs used in the clinical practice [8]. Nevertheless, it is important to emphasize that no clinical breakpoints have yet been established for them. On the other hand, epidemiological cutoff values (ECVs) have recently been proposed for some fusarioid species complexes [25].

The current treatment of choice for fusarioid keratitis is the use of eye drops containing 5% of natamycin (NAT). Topic AMB (0.3 to 0.5%) can also be successfully used, but it has the disadvantage of not being largely commercialized [26]. Onychomycosis caused by fusarioid spp. may be treated with itraconazole (ITC) or sometimes with terbinafine (systemic) in combination with topic ciclopirox or amorolfine nail lacquers [8].

*Fusarium* taxonomy has been constantly changing over the years and some species have been reassigned to other genera [27]. In fact, a universally accepted taxonomy for the genus is not yet possible [2]. All the fusarioid species of medical interest belong to the *Nectriaceae* family [2,28]. The main *Fusarium* species complexes (SC) with clinical relevance include the *F*. *solani* (FSSC; now *Neocosmospora*), *F*. *oxysporum* (FOSC), *F*. *fujikuroi* (FFSC), *F*. *incarnatum-equiseti* (FIESC), *F*. *sambucinum* (FSAMSC), *F*. *tricinctum* (FTSC), *F*. *chlamydosporum* (FCSC) and *F*. *dimerum* (FDSC; now *Bisifusarium*) [1].

Therefore, the aims of this study were to accurately identify and study genetic relatedness of 60 isolates of fusarioid spp. obtained from cases of superficial infections (keratitis and dermatomycosis) in the Northeast region of Brazil. We also evaluated biofilm formation and performed antifungal susceptibility testing for these strains.

## Methods

### Ethics statement

All clinical and demographic data of the patients were collected in accordance with the Local Research Ethics Committee, from the Onofre Lopes University Hospital, Federal University of Rio Grande do Norte, approved under number 3.769.085. The written consent was waived because of data anonymization and commitment to preserve the identity of the patients and only secondary data obtained from medical records were used.

### Patients demographic and clinical data

A prospective study was performed with 50 patients admitted at the Sector of Ophthalmology, Onofre Lopes University Hospital, Rio Grande do Norte state, Northeast Brazil, between March, 2012 and December, 2022. The patients enrolled in this study belonged to cases of suspected keratitis. In addition, 10 patients with suspected dermatomycoses were forwarded by dermatologists for mycological examination at the Laboratory of Medical and Molecular Mycology, Clinical and Toxicological Analyses Department, Federal University of Rio Grande do Norte.

For the patients with suspected keratitis, demographic and clinical data were recorded as follows: gender, age, occupation, admission date, presence of ocular trauma, affected eye (left, right or both), antifungal treatment, transplantation and clinical outcome. For the patients with suspected dermatomycoses, gender, age and body site affected were recorded.

### Specimen collection and laboratory procedures

Corneal scrapes and swabs were collected from the base and edge of the ulcer by using a sterile surgical blade (# 15 on a Bard–Parker handle) and swab (Absorve-Stuart, Brazil) under topical anesthesia (oxybuprocaine hydrochloride, 0.4% w/v; Latinofarma, Brazil) and slit-lamp magnification. Direct examination of corneal smears was performed after Gram staining and 20% potassium hydroxide (KOH) with optical microscopy (CX21, Olympus, Japan). The smears were inoculated onto the surface of chocolate agar, 5% sheep blood agar, brain heart infusion and thioglycolate (Laborclin, Brazil) and incubated at 37°C for bacterial isolation. For fungal isolation, the samples were seeded on Petri dishes containing Sabouraud Dextrose Agar (SDA), supplemented with 50 g/L chloramphenicol (Mumbai, Maharashtra, India), incubated at 25°C-30°C and 37°C and discarded after 3–4 weeks if there was no growth [13]. Skin and nail scrapings were treated with 20% KOH during 30 min for direct examination. The samples were inoculated on the surface of Petri dishes containing SDA, supplemented with 50 g/L chloramphenicol (Mumbai, Maharashtra, India) and the plates incubated at 25–30°C, for a period of up to 4 weeks before being considered negative [29]. Colonies showing macro and micromorphology compatible with *Fusarium* and related genera (morphology, pigmentation, types of conidia formed, etc.), were further identified with molecular biology techniques at the Westerdijk Fungal Biodiversity Institute, Utrecht, the Netherlands.

### DNA extraction

Isolates were grown on Malt Extract Agar (MEA; 10 g Merck-Darmstadt, Germany malt extract, 20 g Merck agar, 1000 mL H_2_O), incubated at 24°C, using a 12/12 h photoperiod (dark/light), under near UV light. Genomic DNA was extracted using the Wizard Genomic DNA purification Kit (Promega Corporation, Madison, WI, USA), following the manufacturer’s instructions [2].

### PCR assay and DNA sequencing

Polymerase chain reaction amplifications were performed on a GeneAmp PCR System 9700 (Applied Biosystems, Foster City, CA, USA) or a 2720 thermal cycler (Applied Biosystems, Foster City, CA, USA). PCR amplification reactions had a total volume of 12.5 μL and contained 0.1 μL Taq DNA polymerase (5 U/μL BIOTAQ DNA Polymerase, BioLine, Germany), 1.25 μL PCR buffer (10× NH_4_ reaction buffer, BioLine, Germany), 0.5 μL MgCl_2_ (50 mM, BioLine, Germany), 0.5 μL dNTP mix (10 mM, BioLine, Germany), 0.7 μL di-methyl sulfoxide (DMSO, Sigma-Aldrich, Germany), 0.25 μL of each primer (10 μM), and 1 μL of DNA template [30]. Partial sequences of two genes were amplified, as follows: translation elongation factor 1-α (*TEF1-α*) using the EF1 and EF2 primers [31] and RNA polymerase II second largest subunit (*RPB2*) using the 5F2 and 7CR primers [32,33]. Cycling conditions for *TEF1-α* were those of O’Donnell et al. [34] and for *RPB2*, those of Liu et al. [33]. The amplified fragments were purified with Sephadex G-50 fine (GE Healthcare Bio-Sciences AB, Uppsala, Sweden), sequenced using the BigDye Terminator v. 3.1 Cycle Sequencing Kit (Applied Biosystems, Foster City, CA, USA), and subsequently analyzed onto the ABI3730xL Genetic Analyzer platform (Applied Biosystems, Foster City, CA, USA), according to the manufacturer’s recommended protocols [30]. Consensus sequences for each marker were assembled in Geneious Prime v. 2023.2.1 [35] or SeqMan Pro v. 15.3.0 (DNASTAR, Madison, WI, USA) software. Nucleotide sequences were submitted for BLAST analysis, at the NCBI website (http://www.ncbi.nlm.nih.gov) for preliminary species identification. Only sequences deposited in GenBank showing high similarities with our query sequences and an E-value lower than 10^−5^ were used for comparisons. Sequences generated in this study were deposited in GenBank under accession numbers OR552632 to OR552693 (*TEF1-α*) and OR557683 to OR557744 (*RPB2*) (Table 1).

**Table 1:** Identification and biofilm formation of *Fusarium* and *Neocosmospora* spp. Demographic, clinical, and antifungal treatment data of 60 patients diagnosed with keratitis and dermatomycoses from March 2012 to December 2022, in Rio Grande do Norte, Northeastern Brazil.

Patient	Age	Gender	Occupation	Clinical sample	Eye affected	Trauma	Corneal transplant	Antifungal treatment	Clinical outcome	Strain number	Final identification	Accession number (*RPB2*)	Accession number (*TEF*-1α)	Biofilm formation (O.D._450nm_)
Patient 1	68	M	N.I.^a^	Corneal swab	Left	N.I	Yes	KTC^b^ (O^e^) + NAT^d^ (TP^f^)	No clinical improvement	LMMM^h^ 130	*Neocosmospora falciformis*	OR557683	OR52632	0.40 ± 0.01
Patient 2	68	M	N.I.	Corneal swab	Left	N.I	Yes	AMB^c^ (TP) + NAT (TP)	Clinical improvement	LMMM 405	*N*. *falciformis*	OR557686	OR552635	0.20 ± 0.01
Patient 3	42	M	Mechanic	Corneal scrapings	Right	N.I	Yes	AMB (IV^g^)	Clinical improvement	LMMM 439	*N*. *falciformis*	OR557688	OR552637	0.10 ± 0.01
Patient 4	44	F	Waiter	Corneal scrapings	Left	N.I.	Yes	-	Vision loss	LMMM 442	*N*. *falciformis*	OR557690	OR552639	0.16 ± 0.01
Patient 5	58	F	N.I.	Corneal swab	Right	N.I.	Yes	AMB (TP) + KTC (O)	No clinical improvement	LMMM 443	*N*. *falciformis*	OR557691	OR552640	0.09 ± 0.01
Patient 6	41	M	Agriculturist	Corneal scrapings	Left	N.I.	Yes	AMB (TP)	No clinical improvement	LMMM 686	*N*. *falciformis*	OR557693	OR552642	0.03 ± 0.01
Patient 7	59	M	Agriculturist	Corneal scrapings	Right	No	Yes	AMB (TP) + KTC (O)	No clinical improvement	LMMM 689	*N*. *falciformis*	OR557695	OR552644	0.02 ± 0.01
Patient 8	52	M	N.I	Corneal scrapings	Right	N.I	Yes	AMB (TP) + KTC (O)	No clinical improvement	LMMM 692	*N*. *falciformis*	OR557697	OR552646	0.19 ± 0.02
Patient 9	57	M	Agriculturist	Corneal scrapings	Right	Handling of beans	Yes	AMB (TP)	Clinical improvement	LMMM 693	*N*. *falciformis*	OR557698	OR552647	0.13 ± 0.01
Patient 10	29	M	Agriculturist	Corneal scrapings	Right	Yes	No	AMB (TP)	Clinical improvement	LMMM 694	*N*. *falciformis*	OR557699	OR552648	0.11 ± 0.01
Patient 11	45	M	N.I.	Corneal scrapings	Right	No	Yes	-	No clinical improvement	LMMM 697	*N*. *falciformis*	OR557700	OR552649	0.11 ± 0.01
Patient 12	25	M	N.I.	Corneal scrapings	Right	N.I.	N.I.	-	-	LMMM 930	*N*. *falciformis*	OR557702	OR552651	0.15 ± 0.01
Patient 13	23	M	Agriculturist	Corneal scrapings	Left	N.I.	No	AMB(IV)	Loss to follow up	LMMM 932	*N*. *falciformis*	OR557704	OR552653	0.04 ± 0.01
Patient 14	4	M	Student	Corneal scrapings	Left	N.I.	No	AMB(IV) + NAT (TP) + KTC (O)	Clinical improvement	LMMM 933	*N*. *falciformis*	OR557705	OR552654	0.20 ± 0.01
Patient 15	30	M	N.I.	Corneal scrapings	Right	No	No	AMB (TP) + KTC (O)	Clinical improvement	LMMM 1053	*N*. *falciformis*	OR557707	OR552656	0.14 ± 0.03
Patient 16	20	M	Agriculturist	Corneal scrapings	Left	Plant	Yes	AMB (IV) + KTC (O)	No clinical improvement	LMMM 1054	*N*. *falciformis*	OR557708	OR552657	0.09 ± 0.01
Patient 17	59	F	N.I.	Corneal scrapings	Left	N.I.	Yes	AMB (IV) + KTC (O)	No clinical improvement	LMMM 1057	*N*. *falciformis*	OR557711	OR552660	0.24 ± 0.02
Patient 18	35	M	Logger	Corneal scrapings	Right	No	No	-	Loss to follow up	LMMM 1062	*N*. *falciformis*	OR557713	OR552662	0.27 ± 0.01
Patient 19	28	M	N.I	Corneal scrapings	Right	N.I.	N.I.	AMB (IV)	Clinical improvement	LMMM 1212	*N*. *falciformis*	OR557718	OR552667	0.09 ± 0.01
Patient 20	49	F	N.I.	Corneal scrapings	Right	No	Yes	AMB (IV) + KTC (O)	No clinical improvement	LMMM 1357	*N*. *falciformis*	OR557726	OR552675	0.19 ± 0.01
Patient 21	55	M	Agriculturist	Corneal swab	Right	Grass	Yes	AMB (IV) + KTC (O)	Loss to follow up	LMMM 1405	*N*. *falciformis*	OR557730	OR552679	0.07 ± 0.01
Patient 22	48	M	N.I	Vitreous humor	Right.	N.I	Yes	-	No clinical improvement	LMMM 1439	*N*. *falciformis*	OR557732	OR552681	0.10 ± 0.00
Patient 23	28	M	Bricklayer	Corneal swab	Left	N.I.	N.I.	-	Loss to follow up	LMMM 1521	*N*.*falciformis*	OR557735	OR552684	0.22 ± 0.01
Patient 24	40	M	Bricklayer	Corneal swab	Left	Cement	Yes	AMB(IV) + NAT (TP) + KTC (O)	No clinical improvement	LMMM 1524	*N*. *falciformis*	OR557737	OR552686	0.12 ± 0.01
Patient 25	22	M	Flour maker	Corneal swab	Right	Flour	No	AMB (TP) + NAT (TP)	Clinical improvement	LMMM 1528	*N*. *falciformis*	OR557741	OR552690	0.02 ± 0.01
Patient 26	40	M	Welder	Corneal swab	Left	No	No	AMB(IV) + NAT (TP) + KTC (O)	No clinical improvement	LMMM 1529	*N*. *falciformis*	OR557742	OR552691	0.14 ± 0.01
Patient 27	42	M	N.I.	Corneal swab	Left	Motocycle accident	No	AMB(IV) + NAT (TP) + KTC (O)	No clinical improvement	LMMM 1530	*N*. *falciformis*	OR557743	OR552692	0.11 ± 0,01
Patient 28	34	F	Agriculturist	Corneal scrapings	Right	No	Yes	AMB (TP)	Clinical improvement	LMMM 191	*Neocosmospora keratoplastica*	OR557685	OR552634	0.20 ± 0.01
Patient 29	-	-	**-**	Nail scales	**-**	**-**	**-**	-	-	LMMM 436	*N*. *keratoplastica*	OR557687	OR552636	0.16 ± 0.01
Patient 30	-	-	**-**	Nail scales	**-**	**-**	**-**	-	-	LMMM 444	*N*. *keratoplastica*	OR557692	OR552641	0.15 ± 0.01
Patient 31	57	F	N.I.	Corneal scrapings	Left	N.I.	Yes	AMB (TP) + NAT (TP)	No clinical improvement	LMMM 699	*N*. *keratoplastica*	OR557701	OR552650	0.11 ± 0.01
Patient 32	46	M	Woodworker	Corneal scrapings	Left	N.I	N.I.	AMB (IV)	Loss to follow up	LMMM 1067	*N*. *keratoplastica*	OR557715	OR552664	0.14 ± 0.01
Patient 33	22	M	Student	Corneal scrapings	Right	N.I.	N.I.	-	-	LMMM 1068	*N*. *keratoplastica*	OR557716	OR552665	0.12 ± 0.01
Patient 34	59	F	**-**	Toenail scales	**-**	**-**	**-**	-	-	LMMM 1263	*N*. *keratoplastica*	OR557720	OR552669	0.21 ± 0.01
Patient 35	46	M	**-**	Toenail scales	**-**	**-**	**-**	-	-	LMMM 1311	*N*. *keratoplastica*	OR557724	OR552673	0.16 ± 0.01
Patient 36	60	F	**-**	Fingernail scales	**-**	**-**	**-**	-	-	LMMM 1376	*N*. *keratoplastica*	OR557727	OR552676	0.12 ± 0.00
Patient 37	67	F	**-**	Foot intertidigital space	**-**	**-**	**-**	-	-	LMMM 1379	*N*. *keratoplastica*	OR557728	OR552677	0.13 ± 0.01
Patient 38	55	M	N.I	Corneal scrapings	Right	Beach sand	N.I.	AMB (IV)	Loss to follow up	LMMM 1461	*N*. *keratoplastica*	OR557733	OR552682	0.08 ± 0.01
Patient 39	56	M	N.I.	Corneal swab	Right	Ceiling debris	Yes	AMB (TP) + NAT (TP)	Loss to follow up	LMMM 1523	*N*. *keratoplastica*	OR557736	OR552685	0.11 ± 0.01
Patient 40	19	M	N.I.	Corneal swab	Left	Plaster powder	No	AMB (TP) + NAT (TP)	Clinical improvement	LMMM 1527	*N*. *keratoplastica*	OR557740	OR552689	0.10 ± 0.00
Patient 41	14	F	N.I.	Corneal scrapings	Right	N.I	N.I.	-	-	LMMM 687	*Neocosmospora suttoniana*	OR557694	OR552643	0.09 ± 0.02
Patient 42	39	M	Cashew nuts worker	Corneal scrapings	Left	N.I.	N.I.	-	-	LMMM 1056	*N*. *suttoniana*	OR557710	OR552659	0.13 ± 0.01
Patient 43	27	M	N.I	Corneal scrapings	Right	N.I	Yes	AMB(TP) + NAT (TP)+ KTC (O)	Clinical improvement	LMMM 1066	*N*. *suttoniana*	OR557714	OR552663	0.18 ± 0.01
Patient 44	41	M	N.I.	Corneal scrapings	Right	Grass	Yes	AMB (TP) + NAT (TP)	Clinical improvement	LMMM 1355	*N*. *suttoniana*	OR557725	OR552674	0.45 ± 0.02
Patient 45	42	M	**-**	Corneal swab	**-**	**-**	**-**	-	-	LMMM 1531	*N*. *suttoniana*	OR557744	OR552693	0.20 ± 0.01
Patient 46	42	F	**-**	Toenail scales	**-**	**-**	**-**	-	-	LMMM 1276	*Neocosmospora gamsii*	OR557721	OR552670	0.05 ± 0.00
Patient 47	35	F	**-**	Fingernail scales	**-**	**-**	**-**	-	-	LMMM 1277	*N*. *gamsii*	OR557722	OR552671	0.13 ± 0.01
Patient 48	48	M	N.I.	Corneal scrapings	Right	No	Yes	AMB (TP)+ KTC (O)	Clinical improvement	LMMM 440	*Fusarium annulatum*	OR557689	OR552638	0.21 ± 0.01
Patient 49	63	F	Agriculturist	Corneal scrapings	N.I.	N.I.	Yes	AMB(IV) + NAT (TP) + KTC (O)	No clinical improvement	LMMM 931	*F*. *annulatum*	OR557703	OR552652	0.20 ± 0.01
Patient 50	64	M	N.I	Corneal scrapings	Right	N.I	N.I.	-	Loss to follow up	LMMM 1058	*F*. *annulatum*	OR557712	OR552661	0.11 ± 0.01
Patient 51	69	M	Agriculturist	Corneal scrapings	Left	Tree branch	N.I.	AMB (IV)	Clinical improvement	LMMM 1208	*F*. *annulatum*	OR557717	OR552666	0.17 ± 0.01
Patient 52	38	M	**-**	Toenail scales	**-**	**-**	**-**	-	-	LMMM 1258	*F*. *annulatum*	OR557719	OR552668	0.14 ± 0.01
Patient 53	65	M	**-**	Right plantar region	**-**	**-**	**-**	-	-	LMMM 1309	*F*. *annulatum*	OR557723	OR552672	0.16 ± 0.01
Patient 54	55	M	N.I.	Corneal swab	Left	Tree branch	Yes	KTC(O) + NAT (TP)	Loss to follow up	LMMM 1382	*F*. *annulatum*	OR557729	OR552678	0.15 ± 0.01
Patient 55	-	F	**-**	Corneal swab	**-**	**-**	**-**	-	-	LMMM 1525	*F*. *annulatum*	OR557738	OR552687	0.15 ± 0.01
Patient 56	26	M	N.I	Corneal swab	Left	N.I.	No	NAT (TP)	Clinical improvement	LMMM 1526	*F*. *annulatum*	OR557739	OR552688	0.15 ± 0.01
Patient 57	63	M	N.I.	Corneal scrapings	Left.	N.I.	No	AMB (TP) + NAT (TP)	Clinical improvement	LMMM 690	*Fusarium nirenbergiae*	OR557696	OR552645	0.12 ± 0.01
Patient 58	46	F	N.I.	Corneal scrapings	Right	N.I.	No	-	Loss to follow up	LMMM 934	*F*.*nirenbergiae*	OR557706	OR552655	0.15 ± 0.03
Patient 59	29	F	N.I	Corneal scrapings	Both	No	N.I.	AMB (IV) + KTC (O)	Clinical improvement	LMMM 1055	*F*.*nirenbergiae*	OR557709	OR552658	0.15 ± 0.01
Patient 60	37	F	N.I.	Corneal swab	Both	No	Yes	KTC(O) + NAT (TP)	Clinical improvement	LMMM 1520	*F*. *nirenbergiae*	OR557734	OR552683	0.11 ± 0.01

_N. I._^a^_= not informed. KTC_^b^_= Ketoconazole. AMB_^c^_= amphotericin B. NAT_^d^_= natamycin. O_^e^_= oral. TP_^f^_= topical. IV_^g^_= intravenous. LMMM_^h^_= Laboratory of Medical and Molecular Mycology._

### Phylogenetic analyses

Sequences of the individual markers, including introns, were aligned with ex-type and reference sequences via MAFFT v. 7.110 [36] using default parameters and adjusted manually, when necessary. Alignments were analyzed using Maximum Likelihood (ML) and Bayesian Inference (BI). For the ML analyses, concatenated phylogenies, where each marker was treated as a separate partition, were determined using IQ-TREE v. 2.1.2 [37] with ultrafast bootstrapping [38] for estimation of branch support. Additional ML analyses were performed using RAxML v. 8.2.12 [39]. To assess the robustness of the analyses, the Bootstrap support (BS) was determined automatically by the software using default parameters. The BI analysis was performed using MrBayes v. 3.2.6 [40] on the CIPRES science gateway portal [41], using four Markov chain Monte Carlo (MCMC) chains starting from a random tree topology.

### Morphological studies

In order to check if the molecular identification was compatible with phenotypic characteristics of each species, micromorphological characters were observed after 7–10 days of incubation on carnation leaf agar (CLA; sterile leaf pieces, 20 g Merck agar, 1000 mL H_2_O) [42]. All microscopic characters were examined using sterile water as a mounting medium with a Zeiss Axioskop 2 compound light microscope, including the shape of macroconidia produced in sporodochia, the shape and mode of formation of microconidia and chlamydospores on CLA. To facilitate the comparison of relevant micro- and macroconidial features, composite photo plates were assembled from separate photo micrographs using Adobe Photoshop CC [2].

### Biofilm formation of *Fusarium* spp. and related genera

Biofilm formation assays were performed according to Sav et al. [24] with some modifications. The clinical isolates were previously cultivated in Potato Dextrose Agar (PDA; Merck, Darmstadt, Germany) slants at 25–30°C, for 7 days. Then, 1 mL of phosphate buffered saline (PBS) was added to the cultures and further vortexed to make a conidial suspension. Next, the cell suspension of each isolate was adjusted to a final concentration of 1 × 10^6^ conidia/mL) using a hemocytometer. Subsequently, 200 μL of the suspension was transferred to flat bottom, 96 well microtiter plates and incubated for 2 h at 37°C for the adhesion phase in quintuplicate (test wells). As controls, eight wells of each microtiter plate were handled similarly, except that no conidial suspension was added. Following the adhesion phase, cell suspensions were aspirated and each well was washed twice with 150 μL of PBS to remove loosely adherent cells. A total of 200 μL of brain-heart infusion broth (BHIB; Difco Franklin Lakes, NJ, USA) supplemented with glucose (0.25%) was added to each of the washed wells and incubated at 37°C in a shaker incubator at 100 rpm. Biofilms were allowed to develop for 24 h and quantified by a crystal violet assay. Briefly, the biofilm-coated wells of the microtiter plates were washed trice with 150 μL of PBS and then air dried for 30 min. Subsequently, each of the washed wells was stained with 200 μL of 1% aqueous crystal violet solution for 30 min. Next, each well was washed trice with 200 μL sterile distilled water and immediately distained with the addition of 200 μL of 33% acetic acid, for approximately 30 min until complete crystal violet solubilization. Subsequently, 100 μL of the distaining solution was transferred to a clean well and the amount of crystal violet stain in the referred solution was quantified with a microtiter plate reader (SpectraMAX 340 Tunable Microplate Reader; Molecular Devices Ltda., San Jose, CA, USA) at 450 nm. The absorbance values for the controls were subtracted from the values of the test wells to minimize background interference. The isolates were classified according to terciles distribution as follows: 0.01 ≤ OD_450nm_ ≤ 0.14, as weak; 0.15 ≤ OD _450nm_ ≤ 0.29, as moderate; 0.30 ≤ OD_450nm_ ≤ 0.45, as strong biofilm producers.

### Antifungal susceptibility testing

Solutions of KTC, ITC and AMB were diluted in RPMI 1640 (Roswell Park Memorial Institute; Angus buffers and Biochemical, Niagara Falls, NY, USA) buffered with 3-(N-morpholino) propanesulfonic acid (MOPS) to pH 7.0. Antifungal drugs were diluted serially in 10 different concentrations, namely: KTC (Sigma Chemical Corporation, St. Louis, MO, USA) 0.0313–16 μg/mL; ITC (Pfizer Incorporated, New York, NY, USA), and AMB (Sigma Chemical Corporation, St. Louis, MO, USA) 0.0313–16 μg/mL. The inocula of all isolates tested were obtained from 48 to 72 h cultivation in PDA at 35°C followed further incubation at 25 to 28°C for 7 days. One milliliter of saline solution was added to each culture to prepare a conidial suspension. The suspension was adjusted to an optical density that ranged from 0.15 to 0.17 at 530 nm. Then, a serial dilution was made in RPMI (1:50), in order to obtain a final concentration of 0.4 to 0.5 × 10^4^ cells/mL. Susceptibility to antifungal agents was evaluated by broth microdilution, as follows: Aliquots of 100 μL of the final inoculum solution were dispensed in microtiter plates of 96 wells containing 100 μL of various concentrations of the tested drugs. Finally, the plates were incubated at 37°C and test reading taken after 72 h incubation. MIC was defined for KTC to the lowest drug concentration which showed approximately 50% reduction in turbidity as compared to the positive control well. For AMB and ITC, the MIC was defined as the lowest concentration able to inhibit any growth visually perceptible [43].

### Statistical analysis

Data were analyzed using the statistical software GraphPad Prism version 8.0 (GraphPad Software, San Diego, CA, USA). Results were presented as mean ± standard deviation, and differences were analyzed by the Mann–Whitney test. For all the analyses, *P* was considered a default value of 0.05 and the confidence interval of 95%.

## Results

### Clinical, demographic and epidemiological characteristic of the patients

A total of 50 patients with suspected keratitis were enrolled in this study between March 2012 and December 2022. The majority of individuals were male (n = 37; 74%), while 13 (26%) of them were female. Patient´s age ranged from four to 69 years old, average of 42.4 ± 16 years old, median of 42 years old and mode of 68 years old. Thirteen patients (26%) reported previous trauma of the eye, while 10 (20%) denied it, and the information was unknown for 27 (54%) of them. For the patients who reported trauma, most of the reported cases were related to injury with plant material or soil. The majority of patients were agricultural or plant related workers (approximately 67% of reported cases). Right or left eyes were relatively equally affected (50% versus 40%, respectively), while in 4% of the cases, both eyes were infected with fusarioid spp. Corneal transplant was performed in 26 patients (52%), while for 24 patients (12 each), the transplant was either not performed or nor informed (48%). The patients received the following antifungal drugs regimen: 10 of them (20%), AMB only. Ten (20%), a combination of AMB and KTC. Seven (14%), a combination of AMB and NAT. Six of them (12%) received a combination of AMB, NAT and KTC. Three patients (6%) each received either a combination of KTC and NAT or antibacterial agents only (totalizing 12%), while treatment information is missing for 10 (20%) of the patients. Eighteen patients showed clinical improvement (36%), while 16 of them (32%) did not show visual improvement after antifungal treatment, corneal transplantation or surgery. Loss of patient follow up/missing information was observed in 32% of patients. Only two patient had bacterial eye infections together with fusarioid keratitis, as follows: patient 33 (*Acinetobacter baumannii*) and 38 (*Staphylococcus aureus*). Regarding patients with dermatomycoses, 10 individuals were included, being five females (50%) and three males (30%). Patient’s age ranged from 38 to 67 years old, average of 51.5 ± 12.7 years old, median of 52.5 years old. Most of the onychomycosis lesions were found on the toenails (4; 40%), followed by fingernails (2; 20%). *Tinea pedis* accounted for 20% of cases, being one of them in the plantar and the other one in the interdigital region of the feet. Clinical and demographic data is missing for two (20%) patients. The collection period was between August, 2017 to September, 2018 (Table 1).

### Phenotypic preliminary identification of the strains used in the present study

A total of 60 positive cultures were found (50 from corneal smears and 10 from nail or epidermal scales). All the strains produced fusarium-like colonies, macro and micromorphology with the presence of sickle-shaped septate macroconidia and/or smaller, usually non-septate microconidia.

### Molecular identification and phylogenetic analysis of the strains used in the present study

**Fig 1:**
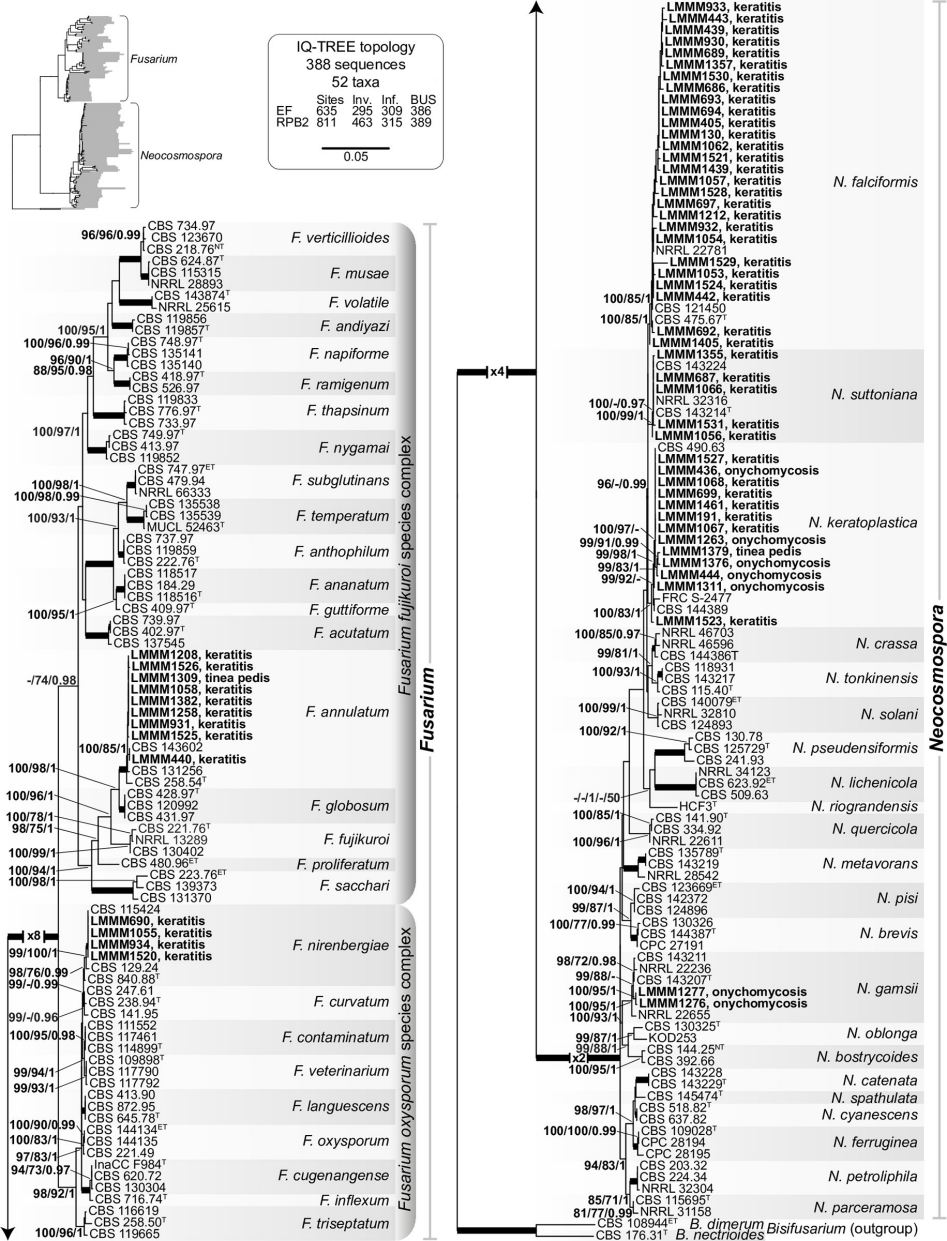
(IQ-TREE-ML) phylogeny inferred from combined *TEF1*-α and *RPB2* datasets of members of *Neocosmospora* spp., *Fusarium fujikuroi* species complex (FFSC) and *Fusarium oxysporum* species complex (FOSC). Support values at the branches indicate IQ-TREE bootstrap (BS)/RAxML-BS/Bayesian posterior probability values (PP) above 95/70/0.95, with thickened branches indicating full support (BS = 100 and PP = 1). Inset shows the composition of the datasets (total, invariable, and informative sites, respectively; followed by Bayesian unique site patterns (BUS). The scale bar indicates expected changes per site. Keratitis and dermatomycosis clinical isolates from Rio Grande do Norte, Northeast Brazil isolated in this study are shown in bold font. The tree is rooted to *Bisifusarium nectrioides* (CBS 176.31) and *Bisifusarium dimerum* (CBS 108944). Ex-epitype, ex-neotype and ex-type strains are indicated with ^ET^, ^NT^, and ^T^, respectively. LMMM stands for Laboratory of Medical and Molecular Mycology.

For molecular species identification, DNA fragments of *TEF1-α* and *RPB2* were amplified and the sequences obtained were lodged in the GenBank sequence database (NCBI, http://ncbi.nlm.nih.gov) using BLAST, to compare gene sequences. Phylogenetic analyses confirmed the species identification of 47 isolates as belonging to the genus *Neocosmospora* (78.33%), nine of them to the FFSC (15%) and four isolates to the FOSC (6.66%). The clinical isolates were identified as follows: 27 of them as *N*. *falciformis* (45%), all of them isolated from keratitis cases; thirteen isolates as *N*. *keratoplastica* (21.66%), of which seven were from keratitis cases and six from dermatomycosis. Nine isolates were identified as *F*. *annulatum*, seven from keratitis and two from dermatomycosis (15%). Five isolates were identified as *N*. *suttoniana* (8.33%), all of them obtained from corneal smears. Four isolates (6.6%) of *F*. *nirenbergiae* obtained from keratitis cases were found. Finally, two (3.3%) isolates obtained from onychomycosis were identified as *N*. *gamsii*. It is important to emphasize that preliminary BLAST searches had either identified some strains belonging to *Neocosmospora* as *F*. *solani sensu stricto* or were not able to distinguish the species within the *Neocosmospora* clade. In addition, *F*. *nirenbergiae* strains were misidentified as *F*. *oxysporum sensu lato*, while *F*. *annulatum* strains were identified as *F*. *proliferatum*. These uncertainties were solved with phylogenetic analyses (Fig 1).

### Morphological studies

**Fig 2:**
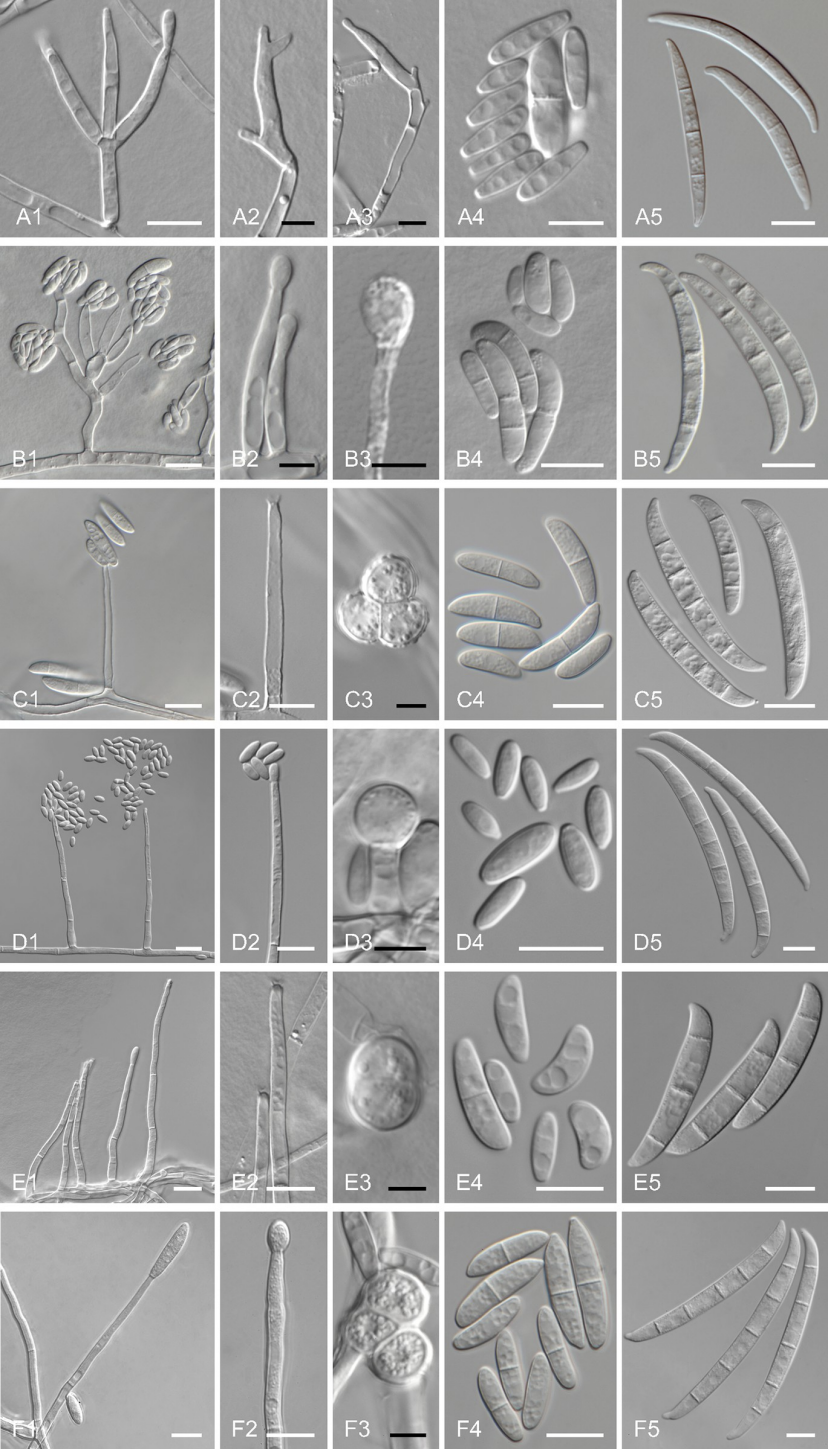
Key morphological features of *Fusarium annulatum* LMMM 1382 (A1 to A5), *Fusarium nirenbergiae* LMMM 934 (B1 to B5), *Neocosmospora falciformis* LMMM 405 (C1 to C5), *Neocosmospora gamsii* LMMM 1277 (D1 to D5), *Neocosmospora keratoplastica* LMMM 1068 (E1 to E5) and *Neocosmospora suttoniana* LMMM 1066 (F1 to F6) after 7–10 days of incubation of the isolates on CLA at 25°C. 1 = Conidiophores. 2 = Phialides. 3 = Chlamydospores (except for *F*. *annulatum =* phialide). 4 = Microconidia and 5 = Macroconidia. Scale bars: black = 5 μm, white = 10 μm.

Micromorphological observations revealed that all the strains grown on CLA had fungal structures compatible with each species, as identified by DNA sequencing and phylogenetic analysis (Fig 2).

### Evaluation of biofilm formation in *Fusarium* spp. and related genera

**Fig 3:**
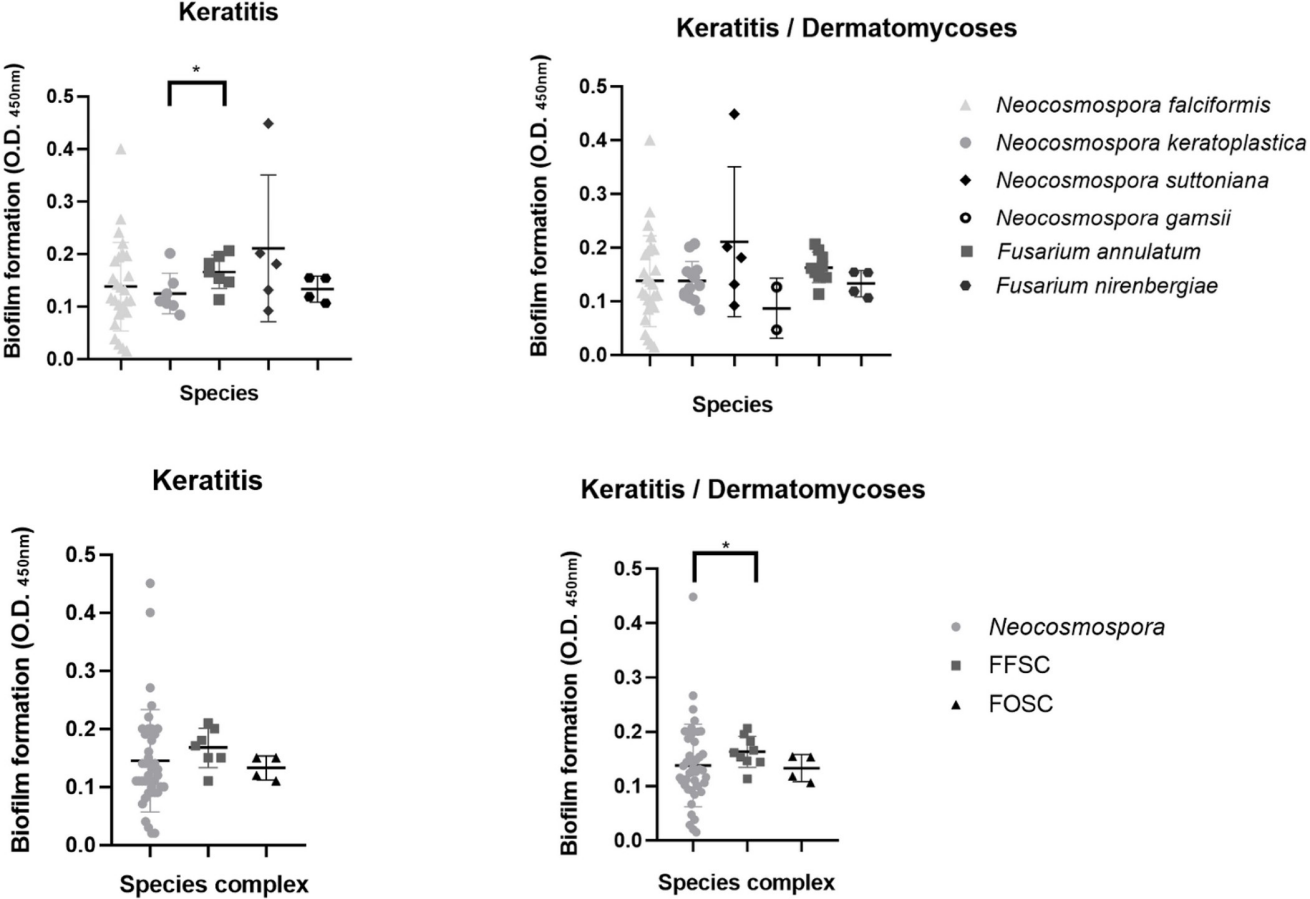
Biofilm formation of *Fusarium* and *Neocosmospora* spp. induced after incubation of cells in 96-wells microtiter plates containing BHI medium at 37°C, for 48 h. Each bar represents mean ± standard deviation for all isolates of the same species. *P < 0.05.

There was a large variation in biofilm biomass among all the clinical isolates evaluation. In *N*. *falciformis*, the OD_450nm_ ranged from 0.02±0.01 (LMMM1528) to OD_450nm_ of 0.40 ± 0.01 (LMMM 130). The average OD_450nm_ for all the strains tested was 0.14 ± 0.08. With regard to tercile analysis, 17 isolates (62.96%) were considered weak, nine moderate (33.33%), while one (3.7%) isolate was a strong biofilm producer. In *N*. *keratoplastica*, the OD_450nm_ ranged from 0.08±0.01 (LMMM1461) to OD_450nm_ of 0.21 ± 0.01 (LMMM1263). The average OD_450nm_ for all the strains tested was 0.14 ± 0.04. Most isolates were weak biofilm producers (8; 61.53%), while five of them (38.46%) were moderate biofilm producers. Eight-six of all *F*. *annulatum* keratitis isolates (n = 6) were moderate biofilm producers. When the strains obtained from nails and skin were included in the analysis, the percentage of moderate biofilm producers is still higher than the ones considered of low biofilm producers (66.66% versus 33.33%, respectively). The average OD_450nm_ for all the strains tested was 0.16 ± 0.03, ranging from 0.11 ± 0.01 (LMMM1058) to 0.21 ± 0.03 (LMMM440). Interestingly, the clinical isolate that showed the highest biofilm formation belonged to *N*. *suttoniana* (LMMM1355; OD_450nm_ 0.45 ± 0.02). However, there was a large variation in biofilm formation among the strains of this species, with 40% of them classified as weak and moderate biofilm producers, each. A similar trend of variation was observed among isolates of *F*. *nirenbergiae* (50% each of weak and moderate producers), with an average OD_450nm_ of 0.13 ± 0.02. The two isolates identified as *N*. *gamsii* were weak biofilm producers (Table 1 and Fig 3). The only statistically significant comparison was observed between *F*. *annulatum* and *N*. *keratoplastica* obtained from keratitis (0.17 ± 0.03 versus 0.12 ± 0.04, respectively; *P* = 0.04). This difference was also observed when comparing all strains (keratitis and dermatomycosis) from each SC (or *Neocosmospora*): FFSC versus *Neocosmospora* (0.16 ± 0.01 versus 0.14 ± 0.01, respectively; *P* = 0.04; Fig 3).

### Antifungal susceptibility testing for *Fusarium* spp. and related genera

MIC ranges, MIC50, MIC90, MIC97.5 and geometric means (GMs) for all the antifungal drugs tested are displayed in Table 2. Interestingly, only a few strains had increased MICs if we consider the MIC97.5 as a cut-off value (equivalent to the ECV). For KTC, one isolate of *N*. *falciformis* and 2 isolates of *N*. *keratoplastica* showed MICs above 16 μg/mL. For all the other strains tested, the MICs were ≤ MIC97.5. Nevertheless, several strains showed AMB MIC = 8 μg/mL which corresponds to a single dilution lower than the MIC97.5.

**Table 2:** Pooled MICs distribution, geometric means, MIC range, MIC50 and MIC90 of 60 clinical isolates of *Fusarium* and *Neocosmospora* spp. obtained from patients diagnosed with keratitis and dermatomycoses, from March 2012 and December 2022, in Rio Grande do Norte, Northeast Brazil, as determined by the CLSI broth microdilution method.

Antifungal drug	Species	MIC^a^ range (μg/mL)	MIC 50	MIC 90	MIC 97.5	MIC geometric mean (μg/mL)	No of isolates tested	No. of isolates with a MIC (μg/mL) of:							
								<0.25	0.5	1	2	4	8	16	>16
Ketoconazole	***Neocosmospora falciformis***	0.0625 to >16	2	16	>16	4.11	27	3	3	1	8	6	2		1
	***Neocosmospora keratoplastica***	0.125 to >16	8	>16	>16	7.28	13	1	2	1	1	1	1	4	2
	***Neocosmospora suttoniana***	0.125 to 16	2	16	16	7.22	5	1			2			2	
	***Neocosmospora gamsii***	0.25 to 4	0.25	4	4	2.12	2		1			1			
	***Fusarium annulatum***	0.25 to 16	1	16	16	4.13	9	1	2	2	1		2	1	
	***Fusarium nirenbergiae***	1 to 8	2	8	8	3.25	4			1	2		1		
Itraconazole	***Neocosmospora falciformis***	0.125 to 4	2	4	4	1.59	27	3	3	7	11	3			
	***Neocosmospora keratoplastica***	0.25 to 8	1	8	8	2.53	13	2	3	3	2		3		
	***Neocosmospora suttoniana***	0.25 to 8	0.5	8	8	2.05	5	1	2	1			1		
	***Neocosmospora gamsii***	1	1	1	1	1	2			2					
	***Fusarium annulatum***	0.25 to 4	1	4	4	1.36	9	1	2	3	2	1			
	***Fusarium nirenbergiae***	0.5 to 8	0.5	8	8	2.75	4		2		1		1		
Amphotericin B	***Neocosmospora falciformis***	0.5 to 8	2	8	8	3.35	27		1	4	9	9	4		
	***Neocosmospora keratoplastica***	0.25 to 16	4	16	16	5.94	13	1		1	2	4	3	2	
	***Neocosmospora suttoniana***	4 to 16	4	16	16	7.20	5					3	1	1	
	***Neocosmospora gamsii***	8	8	8	8	8	2						2		
	***Fusarium annulatum***	0.25 to 16	4	16	16	4.69	9	1		2		4	1	1	
	***Fusarium nirenbergiae***	1 to 4	2	4	4	2.25	4			1	2	1			

MIC^a^ = minimum inhibitory ***concentration***.

## Discussion

Fungal keratitis is a serious problem that may lead to blinding and has been considered an ophthalmology emergency [10,44,45]. It is one of the leading causes of blindness among young people. Currently, there is a call for attention for fungal keratitis to be considered a neglected tropical disease, recognized by the world health organization [46,47]. Estimations indicate approximately 1.5 million new cases per year [48] with a greater prevalence in tropical and subtropical countries, where it accounts for 20 to 60% of microbial keratitis and are frequently related to agricultural-related eye trauma [49,50]. Another remarkable fact is that approximately 10 to 25% of affected patients will require eye surgery, and around 60% of them may develop blindness even after transplantation and antifungal treatment [51,52] and 8–11% of patients having to have the eye removed.

In the present study, most of the patients with keratitis were male (74%) had a median age of 42 years old, worked with plant material or debris, while 26% of them reported trauma in the eye. This agrees with the literature, where this disease has been related to agricultural activities that are usually performed by males with ages ranging from 20 to 40 years old [13,53–55], a demographic more commonly found among tropical and subtropical developing countries. By contrast, case series from developed, temperate nations frequently report contact lens wear as the main risk factor, with higher female prevalence [56–58], for which clinical outbreaks have been previously reported [59,60]. Moreover, we did not encounter any case of fusarioid keratitis related to contact lens wear, which agrees with a previous Brazilian study, where the incidence of this risk factor was only 5% [54].

In the present study, the number of therapeutic keratoplasties was above 50%. This high percentage of necessity for corneal transplant is in agreement with reports from other developing countries [61]. In a study conducted in Sao Paulo, Brazil, therapeutic grafts were needed for 22 patients (54%) with fusarioid keratitis [54]. On the contrary, in the Netherlands, in a series of 89 cases of this infectious disease from 16 different hospitals, only 22% of the patients required corneal transplantation [57].

AMB was the most frequent antifungal drug used to treat the patients with *Fusarium* keratitis, either as a monotherapy or in combination with NAT and/or KTC. It is considered an alternative for keratitis treatment but the eye drop presentation has to be acquired from a compounding pharmacy and its toxicity limits its usage [26]. On the other hand, NAT has been considered the therapy of choice to treat *Fusarium* keratitis, but it does not have effective penetration on the corneal stroma [62]. Topical VRC may also be applied, but may result in increased corneal perforation after keratoplasty, while it is less effective than NAT [52]. Oral KTC and ITC may be successfully used to treat fusarioid keratitis when VRC (a costlier antifungal agent) is not available [61,63].

Despite the low number of cases of dermatomycoses in the present study, our findings agree with the literature, where most of patients are female and the lesions found on the toenails. In a review conducted by Uemura et al. [18] regarding onychomycosis reported from 16 countries, 66.27% of the cases were found in women. Furthermore, in 46.5% of cases, the lesions were found on toenails, whereas in 10.46% of cases, both toenails and fingernails were affected.

Factors associated with higher prevalence of fusarioid onychomycosis on toenails compared to fingernails include larger ungueal size, slower growth rate, less blood flow and constant trauma associated with humidity [64,65].

The vast majority of the isolates in the present study were identified as *Neocosmospora* spp., independently of the body site affected. In fact, a study conducted by Mayayo et al. [66] revealed that the former *F*. *solani* strains were lethal in a murine model of systemic infection, whereas *F*. *oxysporum*, *F*. *verticillioides* and *F*. *proliferatum* were significantly less virulent. *Neocosmospora* strains could have been more frequently isolated from humans due to higher virulence to mammal hosts.

In a study conducted with 44 strains obtained from patients originated from different parts of the globe and collected from several body sites, most of the fusariosis cases were caused by *Neocosmospora* spp., corroborating our findings [67]. The main species isolated were *N*. *keratoplastica* (31.8%), all obtained from dermatomycoses and *N*. *falciformis* (20%), most of them isolated from keratitis. *N*. *keratoplastica* was also the most isolated fusarioid species from 47 cases of onychomycosis in Taiwan (corresponding to almost 60%) [68].

In another case series of keratitis conducted in Taiwan, the main species found were also *N*. *falciformis* (32.6%) and *N*. *keratoplastica* (27.9%), among other species of *Neocosmospora*. Species belonging to the FDSC, FOSC (including one strain of *F*. *nirenbergiae*) and FFSC were also isolated, demonstrating a greater species diversity than the present study [69]. *N*. *falciformis* corresponded to 95% of the isolates obtained from cases of keratitis in the South of India [70]. That was also the main species found in our patients with keratitis.

In a German study with 22 patients with *Fusarium* keratitis, the clinical isolates were identified as belonging to *Neocosmospora*, FFSC and FOSC. The authors described a high prevalence of *N*. *petroliphila* (a species not found in the present study) and other species belonging to the FOSC (27%, each) [56]. Interestingly, a high percentage of *N*. *petroliphila* from keratitis was also described in the Netherlands (another temperate country) [57]. This fact could be somehow related to the variation of fusarioid species distribution in the environment, once they can be isolated from soil and plants [71].

In the present study, there was a high percentage of isolation of *F*. *annulatum* from both keratitis and dermatomycosis. This finding may be a peculiarity of the Northeast region of Brazil. This is a higher prevalence compared to other investigations even if we take into consideration that some of the publications might include strains misidentified as *F*. *proliferatum* when they are in fact *F*. *annulatum*. It is important to emphasize that both species belong to completely different phylogenetic clades within the FFSC [72].

Because of the large variation in the ability to form biofilm among *Neocosmospora* spp. strains, most of the comparisons did not reveal any statistically significant results. For instance, the only two strains that were strong biofilm producers belonged to *N*. *suttoniana* and *N*. *falciformis*, both obtained from keratitis. Conversely, some other *Neocosmospora* spp. strains showed very weak biofilm formation. Interestingly, most *F*. *annulatum* clinical isolates were consistently moderate biofilm producers, and the average values of optical densities for biofilm formation were statistically higher for the FFSC. It is recommended to check in future investigations if these results are not anecdotical.

Currently, there are not many studies comparing biofilm formation among different fusarioid species, specifically when we consider identification to the species level within the complexes. Recently, it has been demonstrated that *N*. *falciformis* may form biofilm on polystyrene plates and that conidial aggregates are important for the adhesion phase, whereas anastomosed and well-developed hyphae embedded in an extracellular matrix are found in mature biofilms [73]. In addition, it has been shown that *N*. *keratoplastica* is able to form AMB-resistant biofilm on intravenous catheters [74].

A limitation in the interpretation of the antifungal susceptibility testing results is related to the lack of established clinical breakpoints for fusarioid spp. In addition, it was not possible to establish an ECV in the present study (despite the fact that we have determined MIC97.5 concentrations), because of the necessity to include at least 100 unrelated strains from three independent laboratories [75]. Recently, ECVs were established for *Neocosmospora* (= FSSC) and FOSC, making it difficult to compare species within each complexes [25].

The results of GMs, MIC50 and MIC90 to AMB in the present study were generally higher than the other case series with *Fusarium* spp. isolated from both keratitis [56,61,69,70] and onychomycosis [18,68]. The systemic AMB used here to treat patients with keratitis from the present study may have somehow resulted in increased MICs.

On the contrary, our ITC MICs were lower than most other studies [25,68]. High MICs were also found for KTC. There are not many studies that include this drug in the panel of antifungal susceptibility testing for fusarioid spp., limiting our comparisons. Nevertheless, oral KTC may be used to treat keratitis caused by these pathogens [9,63,76] and its frequent use in our patients may also explain the increased MICs values found.

It is important to emphasize that the methodologies used to perform this study are the most appropriate and specific ones to evaluate fungi of the genus *Fusarium* and other allied genera. The CLSI methodology used for antifungal susceptibility testing is a reproducible procedure if the protocol is strictly used (culture medium, drug dilutions, test readings and interpretations) [25]. The most important reason to evaluate antifungal susceptibility is when the fungal infection is invasive or cause severe damage to tissue and organs, when acquired drug resistance is suspected, or when the patient is unexpectedly failing therapy. For each of these scenarios, knowing the *in vitro* susceptibility pattern would inform the clinician when making therapeutic choices or changes for the adopted therapy [77].

We have found a high prevalence of fusarioid keratitis in Northeastern Brazil. Our study contributed to the knowledge of the epidemiology of *Fusarium* and allied genera infections and this information could be used to assist in the adoption of strategies to improve prevention, and treatment of fusarioid infections. We emphasize that this is the first study in Northeast Brazil that has made such a deep analysis in this regard, despite our limitations, mainly due to the incomplete information available in the medical records. Fusariosis is an important and neglected disease in this region and other developing and tropical countries, with specific peculiarities, given the high number of cases, increased need for keratoplasty and poor outcome, even after antifungal and surgical treatment. It is an infirmity essentially related to males that work with agriculture or other soil related professions. Species of *Neocosmospora* were the most prevalent etiological agents for both keratitis and dermatomycosis, but a remarkable number of consistently biofilm-producer *F*. *annulatum* isolates was found. Increased MICs (although possibly still of wild-type phenotype) were found against AMB and KTC.

## Supporting information

S1 File Manuscript Data set. (XLSX)
